# Can Non-invasive Brain Stimulation Be Considered to Facilitate Reoperation for Low-Grade Glioma Relapse by Eliciting Neuroplasticity?

**DOI:** 10.3389/fneur.2020.582489

**Published:** 2020-11-12

**Authors:** Hugues Duffau

**Affiliations:** ^1^Department of Neurosurgery, Gui de Chauliac Hospital, Montpellier University Medical Center, Montpellier, France; ^2^Team “Plasticity of Central Nervous System, Stem Cells and Glial Tumors, ” National Institute for Health and Medical Research (INSERM), U1191 Laboratory, Institute of Functional Genomics, University of Montpellier, Montpellier, France

**Keywords:** awake surgery, low-grade glioma, non-invasive brain stimulation, neuroplasticity, transcranial magnetic stimulation

## Introduction

Diffuse low-grade glioma (LGG, i.e., World Health Organization grade II glioma) is a brain primary neoplasm with a constant invasion along the cerebral connectome and with an inevitable malignant transformation, which results in functional worsening and ultimately in the death of the patient (1). To optimize the oncofunctional balance of therapeutic management, namely, to increase both the overall survival and the quality of life (QoL), the purpose is to achieve an early and maximal safe surgical resection, performed until critical neural networks have been identified by means of intraoperative corticosubcortical direct electrostimulation (DES) mapping in awake patients (2). Indeed, despite the lack of randomized controlled trials, complete LGG removal and, when functionally feasible, supracomplete resection [i.e., with an oncological margin around the FLAIR signal abnormality visible on the pre-operative magnetic resonance imaging (MRI)] led to a significant increase in median survival around 15 years (3, 4), while in parallel, electrical-guided surgery allowed a significant reduction of severe persistent deteriorations, even in the so-called “eloquent regions” (5). In fact, mechanisms of neuroplasticity induced by the slow progression of the LGG over the years, explaining why the vast majority of patients do experience only mild (or even no) neurological deficits at diagnosis (usually made because of inaugural seizures), open the door to massive surgical resection in areas deemed to be inoperable in a rigid localizationist view of brain processing, with functional recovery and return to a normal life (6–9). Such a considerable functional redeployment is possible, thanks to an actual meta-networking brain organization, based on dynamic interactions within and between neural circuits subserving sensorimotor, visuospatial, language, cognitive, emotional, and behavioral functions (10).

## Surgery for Low-Grade Glioma and Functional Rearrangement

Nonetheless, despite this major improvement of functional and oncological outcomes following LGG surgery in the two past decades (11), because of its intrinsic diffuse nature, LGG cannot be cured, as evidenced by relapse that may arise even many years after supratotal resection (4). As a consequence, reoperation(s) has been advocated in the event of LGG recurrence, with the aim of reducing again the tumor volume and then decreasing the risk of malignant transformation and prolonging overall survival (12, 13). Interestingly, in a large series with more than 1,000 patients, it has been demonstrated that repeat surgeries were significantly associated with greater survival (3). However, preservation of QoL might seem more uncertain still in case of subsequent surgery, especially when the first resection was interrupted according to individual functional boundaries. Yet, it is worth noting that functional rearrangement has been observed between the first and second intervention, as revealed by intraoperative DES (14, 15). Remarkably, such a functional reorganization, likely elicited by the initial operation itself, the post-operative cognitive rehabilitation (16), and the glioma regrowth, enabled an optimization of the extent of resection while avoiding neurological morbidity (15, 17).

On the other hand, this reconfiguration over the years is seen only in a subgroup of LGG patients, and reoperation did not permit to perform (supra)marginal resection in all cases because of some limitations of neuroplasticity, especially related to the involvement of the “minimal common brain” (18). This “neural core,” with a low interindividual variability (19) and a low plastic potential, is mainly constituted by the input systems (as the visual and somatosensory systems), the output systems (as the pyramidal system), and the subcortical connectivity [as the associative fibers, e.g., the arcuate fasciculus or the inferior fronto-occipital fasciculus (IFOF)]; see a recent probabilistic atlas of brain plasticity (8). Such a limitation of brain adaption in reaction to glioma migration explains why some degrees of cognitive disturbances may be found, despite a normal neurological examination at the standard clinical evaluation, when an objective neuropsychological assessment is performed in LGG patients. These disorders may be identified before any treatment [as semantic impairment if the left IFOF is invaded (20)], or following surgical resection—as subjective empathy changes related to the disconnection of the left cingulum bundle or the right IFOF (21), or lexical access troubles associated to damages of the left inferior longitudinal fasciculus (8). Consequently, neurosurgeons should find the optimal compromise between the dynamics of neural networks allowing compensation after glioma resection and limitations of brain reshaping based on the knowledge of critical cortical hubs and axonal pathways (22, 23). To this end, introducing the fourth dimension to optimize the oncofunctional balance over the years in LGG patients led to the proposal of an original paradigm, that is, to consider a multistage surgical approach. This new concept enables to deal with the individual capacity of the central nervous system to reallocate in reaction to slow glioma progression, at least to some extent (24, 25).

## Non-invasive Brain Stimulation and Neuroplasticity

In this setting, the next step would be to try to promote neural redistribution before reoperation in order to increase the likelihood of achieving an improved extent of resection. This facilitation of brain functional rearrangement seems now possible for several reasons. First, mechanisms underlying neuroplasticity after a first glioma surgery start to be better understood, especially thanks to post-operative neuroimaging studies by means of task-based as well as resting functional MRI (fMRI), which showed a balance between recruitment of perilesional areas and involvement of contralesional homologous regions (26–28). Second, besides fMRI, which is based on the principle of neurovascular coupling, resulting in serious limitations as a low reliability and the impossibility to distinguish critical areas from those that can be compensated following brain insult (29), transcranial magnetic stimulation (TMS) has been proposed for functional mapping in cerebral tumor patients (30). Indeed, by evoking a magnetic field able to bypass the skull, TMS may excite neurons in a suprathresholded manner and then can elicit neuronal activity: this permits to quantify network properties such as excitability and connectivity or to cause a transitory virtual lesion disrupting ongoing task, as DES, but non-invasively (31). However, in a recent investigation that compared navigated repetitive TMS (rTMS) with intraoperative DES in glioma patients, TMS showed only 81.6% sensitivity, 59.6% specificity, 78.5% positive predictive value, and 64.1% negative predictive value for pre-operative language mapping (32), confirming that DES remains the criterion standard. Third, beyond pre-surgical planning, TMS has recently been used to study neuroplasticity before and after tumor surgery. In a recent preliminary experience with 18 patients harboring a left glioma, rTMS language mapping has been achieved before a first and then before a second surgery and confirmed a functional reallocation of language sites, with (i) more “language-negative areas” around the neoplasm during the reoperation in patients in whom critical language areas have been found during the first mapping; (ii) more functional reorganization in slow-growing tumors: in other words, these findings support that eloquent regions can leave the tumor area over time, especially in LGG (33). In agreement with fMRI studies, by generating many language disorders over the right hemisphere, rTMS investigations plead in favor of an active recruitment of the contralesional side to compensate for the glioma growth in the left side (34).

In addition, non-invasive brain stimulation (NBS) techniques by means of rTMS or anodal transcranial direct current stimulation (tDCS), which can actively generate neuromodulation by changing cortical excitability into inhibitory or excitatory direction using magnetic or electric fields, respectively, may enable both to potentiate behavioral performances in healthy volunteers and to facilitate post-lesional neuroplasticity in brain-damaged patients (35, 36). Indeed, repeated sessions of NBS over the healthy brain have significantly improved language functions such as speech, semantic fluency, word retrieval, and verbal learning (37–39). Interestingly, this functional improvement was significantly associated with a modulation of the effective connectivity, especially between the left inferior frontal gyrus and the right insula in verb learning facilitation (40). This is in line with DES mapping, which disrupts behavior by stimulating focally an entry door to a larger circuit (41, 42); even though the effects of NBS are foremost local, neural activity within the whole network is actually affected. For example, regarding movement, the interhemispheric transcallosal inhibitory effects may be modified by applying tDCS to one primary motor cortex, as it can facilitate the contralateral primary motor cortex through potentiation of interhemispheric interactions (43). The same concept has been utilized in patients with cerebral insult, in particular for the therapy of post-stroke aphasia. Indeed, although the actual mechanisms of reorganization elicited by excitatory combined with inhibitory effects of NBS on different nodes of the injured neural networks are still matter of debate, it seems that the complex interactions between the ipsilesional, contralesional, and interhemispheric connectivity may be modulated to facilitate functional compensation (44). For instance, anodal tDCS over the left inferior frontal gyrus resulted in an improvement of speech, naming, and repetition in aphasic patients (45, 46). However, because the effect of NBS is not restricted to the stimulated region but also evokes modifications of the functional connectivity in a wider language circuit (47, 48), beyond excitatory stimulation to perilesional sites, inhibitory low-frequency rTMS has been performed over contralateral homotopic language regions to facilitate post-stroke recovery (49, 50). In the same spirit, NBS has also been used in association with speech therapy to potentiate functional compensation (51).

## Perspectives

Based on these preliminary results in stroke, it could be considered to use NBS in patients who underwent brain surgery, in addition to functional rehabilitation, which is already known to participate in post-operative network rearrangement (16, 52). As mentioned, besides the improvement of QoL, the goal would be to optimize the post-operative functional redeployment and to reopen the door to subsequent surgical resection(s), especially for slow-growing LGG (15). Of note, invasive stimulation has previously been suggested by placing a grid of electrodes over the residual glioma at the end of a first partial resection, in order to perform continuous cortical electrical stimulation simultaneously with behavioral training and then to accelerate plastic reorganization prior to reoperation; however, only five patients have been reported, with a high rate of surgical complications (two infections, one subdural hematoma) due to the invasiveness of this technique (53). Moreover, these findings were not reproduced in the literature. As a consequence, a more reliable and feasible original therapeutic solution in clinical routine might be to develop specific NBS protocols that aim pushing away functional nodes to leave the glioma region. Indeed, contrary to the post-stroke aphasic patients, in whom it has been proposed to use inhibitory rTMS over the right hemisphere, particularly the inferior frontal gyrus, in order to facilitate reinforcement within the left damaged language network (51), the main purpose in brain tumor patients would be to favor the recruitment of the contralateral homologous areas, which have been demonstrated by means of fMRI as playing a pivotal role in recovery following a first surgery (26, 54). In fact, to increase the extent of resection during a reoperation, NBS could be utilized to inhibit the perilesional critical sites and to force them out of the periphery of the surgical cavity, where the tumor removal was interrupted at the end of the first operation because functional boundaries have been reached. In other words, the ultimate goal would be to change the respective weight of the nodes within a large-scale bilateral functional network, or even to modulate the interactions between brain systems—as it has been evidenced that language compensation after surgery for left LGG might involve non-language functions such as attentional resources, i.e., that picture naming recovery was correlated to the recruitment of the right frontoparietal attentional network (28). This means that such an innovative therapeutic strategy can be conceived only in a dynamic metanetworking account of neural processing, breaking with the traditional dogmatic localizationist theory (10); therefore, a stronger link should be built between cognitive neurosciences (as the new field of connectomics), technical advances in neuromodulation tools (as rTMS and tDCS), and elaboration of original management for glioma patients, based on a better understanding and guidance of interactions between tumor progression and brain adaptation. In this spirit, the next question could be to use NBS with the aim of catalyzing neuroplasticity and optimizing the extent of resection for gliomas involving critical neural networks even before the first surgery.

## Author Contributions

The author confirms being the sole contributor of this work and has approved it for publication.

## Conflict of Interest

The author declares that the research was conducted in the absence of any commercial or financial relationships that could be construed as a potential conflict of interest.
